# Suppressed concentration quenching and tunable photoluminescence in Eu^2+^-activated Rb_3_Y(PO_4_)_2_ phosphors for full-spectrum lighting

**DOI:** 10.1038/s41377-024-01607-x

**Published:** 2024-09-20

**Authors:** Ming Zhao, Yeping Ge, Yurong Li, Xiaoyan Song, Zhiguo Xia, Xinping Zhang

**Affiliations:** 1 Institute of Information Photonics Technology, School of Physics and Optoelectronic Engineering, Beijing University of Technology, Beijing, 10083 China; 2 College of Materials Science and Engineering, Key Laboratory of Advanced Functional Materials, Education Ministry of China, Beijing University of Technology, Beijing, 10083 China; 3 State Key Laboratory of Luminescent Materials and Devices, Guangdong Provincial Key Laboratory of Fiber Laser Materials and Applied Techniques, Guangdong Engineering Technology Research and Development Center of Special Optical Fiber Materials and Devices, School of Physics and Optoelectronics, South China University of Technology, Guangzhou, 510641 China

**Keywords:** Inorganic LEDs, Optical materials and structures

## Abstract

Highly efficient inorganic phosphors are desirable for lighting-emitting diode light sources, and increasing the doping concentration of activators is a common approach for enhancing the photoluminescence quantum yield (PLQY). However, the constraint of concentration quenching poses a great challenge for improving the PLQY. Herein, we propose a fundamental design principle by separating activators and prolonging their distance in Eu^2+^-activated Rb_3_Y(PO_4_)_2_ phosphors to inhibit concentration quenching, in which different quenching rates are controlled by the Eu distribution at various crystallographic sites. The blue-violet-emitting Rb_3_Y(PO_4_)_2_:*x*Eu (*x* = 0.1%–15%) phosphors, with the occupation of Rb1, Rb2 and Y sites by Eu^2+^, exhibit rapid luminescence quenching with optimum external PLQY of 10% due to multi-channel energy migration. Interestingly, as the Eu concentration increases above 20%, Eu^2+^ prefer to occupy the Rb1 and Y sites with separated polyhedra and large interionic distances, resulting in green emission with suppressed concentration quenching, achieving an improved external PLQY of 41%. Our study provides a unique design perspective for elevating the efficiency of Eu^2+^-activated phosphors toward high-performance inorganic luminescent materials for full-spectrum lighting.

## Abstract (web summary)

We present a design principle to suppress concentration quenching in Rb_3_Y(PO_4_)_2_:Eu by separating activators and prolonging distance, in which quenching rates are controlled by varying Eu distribution at different sites.

## Graphical abstract

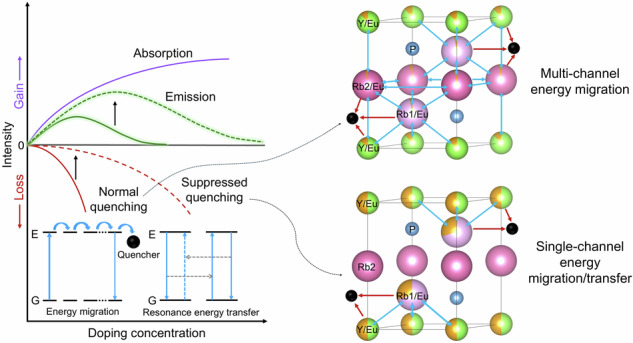

## Introduction

**Fig. 1:**
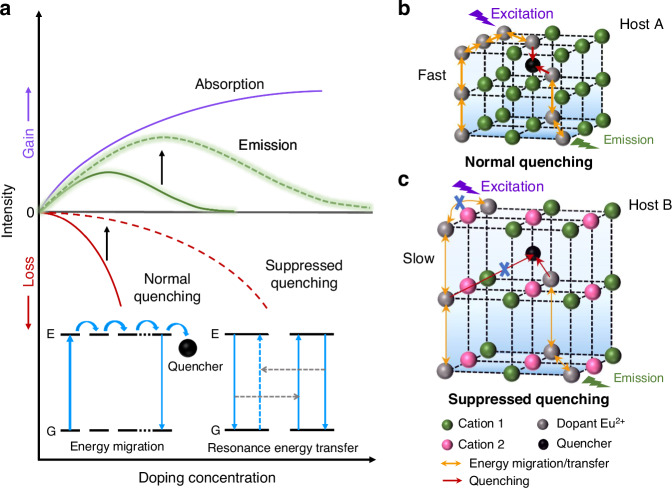
**Schematic diagrams of the concentration quenching in Eu**^**2+**^**-doped phosphors with different types of crystal lattice. a** Effect of Eu^2+^ doping concentration on absorption, emission, and quenching processes in Eu^2+^-activated luminescent materials. The inset shows the two processes leading to quenching, namely long-distance energy migration to quenchers and inter-dopant resonance energy transfer. **b** Schematic representation of normal quenching in heavily Eu^2+^ doped host A with a single cation lattice site and small interionic distances. **c** Schematic representation of suppressed quenching in heavily Eu^2+^ doped host B with multiple cationic lattice sites and large interionic distances. The thin orange arrows denote the reduced rates and probabilities of the related energy migration/transfer processes

Highly efficient inorganic luminescent materials are crucial in realizing energy-efficient white light-emitting diodes (LEDs), which are extensively used in modern lighting, backlit displays, and near-infrared detection technologies^1–8^. These materials typically consist of an inorganic host and one or more doped activators, utilizing the electronic transition of the dopants to covert photon energy. Eu^2+^ is a popular activator in doped luminescent materials due to its electric-dipole-allowed 4*f*-5*d* transition, which enables highly efficient luminescence with tunable emission wavelength and bandwidth^9–11^. The distinctive optical tunability has motivated researchers to develop various Eu^2+^-doped phosphors, with a particular focus on achieving high efficiency, to meet the diverse application requirements of LEDs^12–17^. It is well known that doping Eu^2+^ is achieved by reducing Eu^3+^ with carbon or hydrogen, but the complete reduction is unattainable. Thus, photoluminescence quantum yield (PLQY) can be increased by enhancing the reduction to eliminate luminescence “killer” Eu^3+^, yet the maximum achievable efficiency in the material essentially depends on the doping concentration of Eu^2+^^18–20^. Generally, high doping concentration can provide high-density luminescent centers, thereby improving the efficiency of the luminescent materials. Nevertheless, it can also trigger appreciable quenching process that offsets emission gain and ultimately lead to a falloff in overall emission intensity^21,22^. The effect of Eu^2+^ doping concentration on absorption, emission, and quenching processes in Eu^2+^-activated luminescent materials are shown in Fig. 1a. As a result, the optimal doping concentration of Eu^2+^-activated luminescent materials is typically limited to very low levels, usually 10^−3^ – 10^−2 ^M, beyond which the luminescence intensity decreases rapidly, in consequence essentially limiting the improvement of PLQY of doped phosphors^23^. Therefore, the concentration quenching phenomenon presents a significant challenge in the development of bright Eu^2+^-doped emitters.

Concentration quenching is based on the migration of excitation energy from one activator center to another and eventually to a quencher (the inset of Fig. 1a)^23^. The process of energy migration primarily relies on the distance between the lattice positions occupied by the activator, which would be significantly accelerated at a short distance^24^. In addition, when the doping concentration exceeds a certain threshold, resonance energy transfer would occur in the Eu^2+^-doped phosphors due to electric multipolar intercalation (the inset of Fig. 1a)^25^. The rate (*k*) of the energy transfer process for the dipole-dipole interaction of Eu^2+^ is inversely proportional to distance (*R*), namely *k* ∝ *R*^−6^ ^23,26^. Therefore, the shorter distance between Eu^2+^ ions would facilitate both the energy migration from excited levels to the quenchers and the inter-dopant resonance energy transfer, ultimately leading to a loss in emission intensity. The elevated doping concentration would result in a shorter distance between Eu^2+^ ions, particularly in a host with a single cation lattice site and small interionic distances (Fig. 1b). Based on these principles, it is feasible to minimize the luminescence concentration quenching independent of the type of activators based on a host crystal featuring multiple cationic lattice sites and large interionic distances (Fig. 1c), disfavoring the energy migration/transfer processes.

Therefore, we screened Rb_3_Y(PO_4_)_2_ as the host matrix to verify our design concept due to its structure features. Rb_3_Y(PO_4_)_2_ has three cationic crystallographic sites (Rb1O_7_, Rb2O_12_ and YO_6_) with different interionic distances. The large interionic distances between certain cations may facilitate high concentrations of Eu^2+^ doping with suppressed concentration quenching. Herein, we demonstrated the control of the energy migration/transfer in Rb_3_Y(PO_4_)_2_:Eu^2+^ by strategically changing the occupation of Eu^2+^ at different sites. When the Eu doping concentrations are 0.1%–15%, Eu ions occupy all three cationic sites in Rb_3_Y(PO_4_)_2_, resulting in a blue-violet emission at 427 nm. Due to multi-channel energy migration, rapid concentration quenching occurs at 0.8% Eu concentration. Interestingly, as the Eu doping concentration continues to increase, the samples tune to green emission and inhibit the luminescence quenching until reaching a high concentration of 70%, because of Eu^2+^ preferentially occupying Rb1 and Y sites. The separated Rb1O_7_-Rb1O_7_ and YO_6_-YO_6_ polyhedra with large interionic distances contribute to the reduced rates and probabilities of the energy migration/transfer processes, thereby achieving a high external PLQY (41%) at such high doping concentrations, compared to 10% in the blue-violet-emitting sample. This finding highlights that the targeted selection of a suitable host crystal enables the design of advanced phosphors with high luminescence efficiency, expanding the potential applications in LED technology for full-spectrum lighting.

## Results

### Eu^2+^ site occupation and concentration quenching

**Fig. 2:**
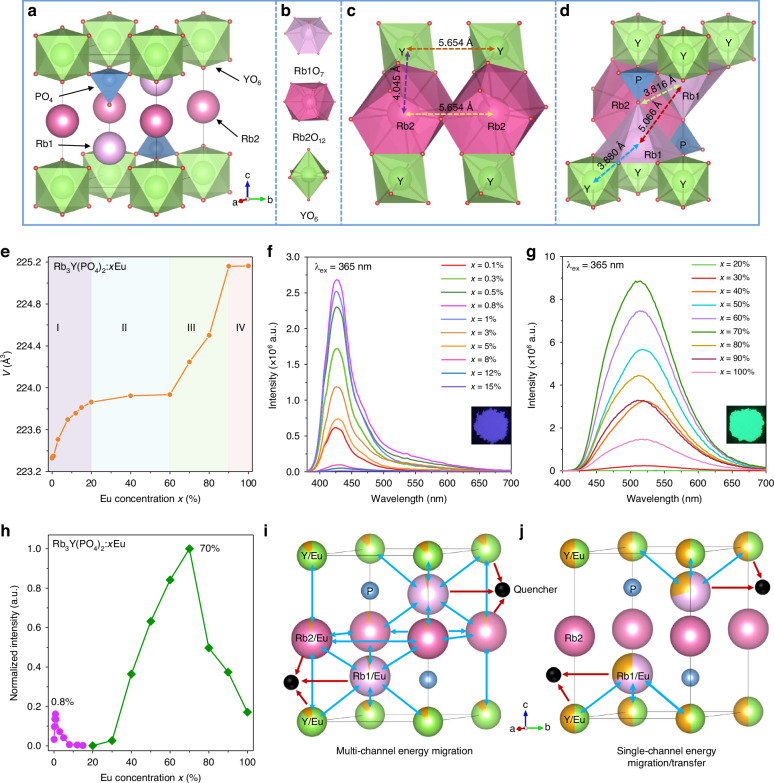
**Crystal structure and concentration quenching analysis of Rb**_**3**_**Y(PO**_**4**_**)**_**2**_**:Eu phosphors. a** Crystal structure overview of Rb_3_Y(PO_4_)_2_. **b** Illustration of Rb1O_7_, Rb2O_12_ and YO_6_ polyhedral subunits. Schematic diagram of the distance between (**c**) Rb2-Rb2, Y-Y, Rb2-Y, and (**d**) Rb1-Rb1, Rb1-Rb2, Rb1-Y atoms. **e** Dependence of cell volume *V* with the Eu concentration *x*. **f**, **g** The emission spectra of Rb_3_Y(PO_4_)_2_:*x*Eu (*x* = 0.1%–100%). The insets are photos of Rb_3_Y(PO_4_)_2_:*x*Eu (*x* = 0.8%, 70%) under a 365 nm UV lamp, which show blue-violet and green luminescence, respectively. **h** Dependence of Eu concentration *x* with the normalized integrated emission intensity. **i** The multi-channel energy migration among Eu(Rb2)-Eu(Rb2), Eu(Rb1)-Eu(Y), Eu(Rb1)-Eu(Rb2)-Eu(Rb1) and Eu(Y)-Eu(Rb2)-Eu(Y) of the blue-violet-emitting Rb_3_Y(PO_4_)_2_:*x*Eu (*x* = 0.1%–15%) phosphors. **j** The single-channel energy migration/transfer among Eu(Rb1)-Eu(Y) of the green-emitting Rb_3_Y(PO_4_)_2_:*x*Eu (*x* = 20%–100%) phosphors

The crystal structure of Rb_3_Y(PO_4_)_2_ host is represented in Fig. 2a. Rb1, Rb2 and P cations are situated between the layers formed by YO_6_ polyhedra. By comparing the ionic radii of Eu and the host cations (Supplementary Table S1), it is theoretically possible for Eu ions to occupy the Rb1, Rb2 and Y cations. These cation sites are coordinated by O^2-^ ions, forming three significantly distinct polyhedra Rb1O_7_, Rb2O_12_ and YO_6_ (Fig. 2b), providing diverse local environments for Eu. Figure 2c, d present the connections between the polyhedra and the distances of the cations. The Rb2O_12_-YO_6_ and Rb1O_7_-Rb2O_12_ polyhedra are connected by shared faces with an interionic distance of 4.045 Å and 3.816 Å. The Rb2O_12_-Rb2O_12_ and Rb1O_7_-YO_6_ polyhedra are connected by a common edge with interionic distances of 5.654 Å and 3.880 Å, respectively. It is worth noting that Rb1O_7_-Rb1O_7_ and YO_6_-YO_6_ polyhedra are not directly connected to each other, and the distances of Rb1-Rb1 and Y-Y are 5.066 Å and 5.654 Å, respectively. Some of the interionic distances are large, exceeding 5 Å, which could inhibit energy migration/transfer of Eu^2+^, resulting in a reduced probability of concentration quenching, as mentioned before^24,27^. Thus, it is possible to achieve high concentrations of Eu doping in the Rb_3_Y(PO_4_)_2_ host and further improve the photoluminescence efficiency. Consequently, we synthesized a series of samples with variable Eu concentrations (0–100%) without reducing the raw material contents, in order to avoid artificial influences on the lattice sites occupied by Eu.

XRD patterns of Rb_3_Y(PO_4_)_2_:*x*Eu (*x* = 0-100%) samples are shown in Supplementary Fig. S1, and all Bragg reflections match well with the simulated pattern of Rb_3_Y(PO_4_)_2_, which crystallizes in the trigonal crystal structure with a space group *P*$\bar{3}$*m*1 (No.164). For the samples with Eu doping concentration *x* ≥ 40%, minor Y_2_O_3_ and Eu_3_(PO_4_)_2_ impurities are observed, but they do not affect the luminescence properties of Eu^2+^ in Rb_3_Y(PO_4_)_2_. The Rietveld refinements of Rb_3_Y(PO_4_)_2_:*x*Eu (*x* = 0-100%) confirmed the structural parameters, as shown in Supplementary Fig. S2 and Supplementary Table S2. According to the trend of the cell volume (Fig. 2e), it can be subdivided into four parts, which will be discussed in detail below. The nonlinear increase in cell volume suggests that the lattice sites occupied by Eu change with increasing Eu doping concentration. To further analyze the substitution mechanisms of Eu ions, the representative samples (*x* = 0%, 8%, 40% and 70%) were selected to refine the Eu site occupancies. The bond lengths and atomic coordinates of these samples are listed in Supplementary Table S3 and Supplementary Table S4. For the sample with low Eu concentration (*x* = 8%), Eu ions predominantly occupy the Y sites with a less portion occupying the Rb1 and Rb2 sites. While for the high Eu doping concentration samples (*x* = 40%, 70%), Eu ions occupy only the Rb1 and Y sites, which could result in different luminescence performances. According to the lattice occupancy, the actual doping concentrations of Eu ions were calculated to be 5.4%, 17.9%, and 36.5% for *x* = 8%, 40% and 70%, respectively. Even though some Eu ions do not enter the lattice, Eu elements are homogeneously dispersed in the particles (Supplementary Fig. S3) and the achieved Eu concentrations are still significant in terms of Eu doping levels. Surprisingly, the samples with such high Eu concentrations (*x* = 40%, 70%) exhibit even higher luminescence intensities with different emission, as discussed below.

Figure 2f, g show the emission spectra of Rb_3_Y(PO_4_)_2_:*x*Eu (*x* = 0.1%–100%) phosphors under 365 nm excitation. For *x* = 0.1%–15%, a blue-violet emission is observed and the optimum Eu^2+^ concentration is 0.8%. Under 365 nm excitation, the external quantum efficiency of the optimized phosphor at *x* = 0.8% is determined to be 10%. Nevertheless, the samples at *x* = 20%–100% present a broadband green emission owing to Eu occupation at different cationic sites. Compared to the samples with low Eu doping concentration, the green-emitting phosphors do not exhibit luminescence quenching until reaching a relatively high concentration of 70%. Moreover, the emission intensity is five times of the blue-violet-emitting phosphor (*x* = 0.8%) (Fig. 2h), and the external quantum efficiency of *x* = 70% phosphor reaches up to 41%, which can be ascribed to the lower probability of energy migration/transfer from the lattice sites occupied by Eu in the green-emitting samples. As shown in Fig. 2i, when Eu ions substitute Rb1, Rb2 and Y cations, energy migration occurs among Eu(Rb2)-Eu(Rb2), Eu(Rb1)-Eu(Y), Eu(Rb1)-Eu(Rb2)-Eu(Rb1) and Eu(Y)-Eu(Rb2)-Eu(Y), leading to rapid luminescence quenching. However, when Eu ions occupy Rb1 and Y sites, due to the lack of direct connection between Eu(Rb1)O_7_-Eu(Rb1)O_7_ and Eu(Y)O_6_-Eu(Y)O_6_ polyhedra, along with the large interionic distances, the energy migration/transfer mainly exists between Eu(Rb1)-Eu(Y) (Fig. 2j), which results in the suppressed concentration quenching thereby realizing a higher luminescence efficiency. The above results are consistent with the previous structural analysis. The emission spectra of the samples at *x* = 8%–20% are displayed in Supplementary Fig. S4 to better observe the variation in the luminescence of the samples from blue-violet (*x* = 15%) to green (*x* = 20%). Due to the low intensity of Eu^2+^ emission in these samples, two characteristic peaks associated with Eu^3+^ are also observed at 538 nm (^5^D_1_ → ^7^F_1_) and 593 nm (^5^D_0_ → ^7^F_1_), implying the presence of Eu^3+^ in the host^28^. To further investigate the characteristics of blue-violet and green luminescence, the samples with *x* = 0.8%, 8%, 40% and 70% are selected for the study below.

### Photoluminescence mechanism

**Fig. 3:**
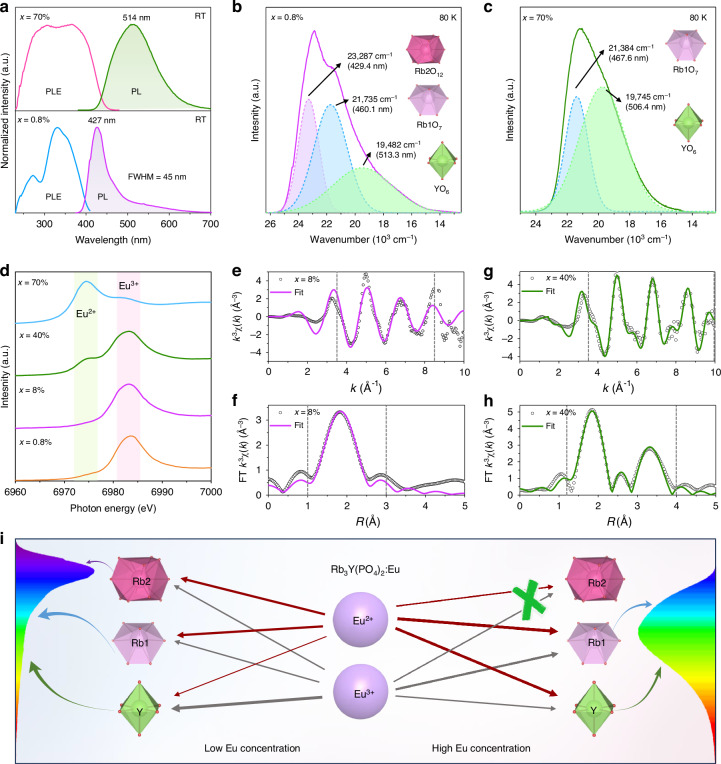
**Photoluminescence properties and mechanism of Rb**_**3**_**Y(PO**_**4**_**)**_**2**_**:Eu phosphors. a** The PL spectra under 365 nm excitation and PLE spectra monitored at corresponding optimal wavelengths of Rb_3_Y(PO_4_)_2_:*x*Eu (*x* = 0.8%, 70%) phosphors at RT. **b**, **c** The low-temperature (80 K) emission spectra and Gaussian fitting results of Rb_3_Y(PO_4_)_2_:*x*Eu (*x* = 0.8%, 70%). **d** Eu *L*_3_-edge XANES spectra of Rb_3_Y(PO_4_)_2_:*x*Eu (*x* = 0.8%, 8%, 40%, 70%). The *k*^3^-weighted Eu *L*_3_-edge EXAFS spectra and corresponding Fourier transform fitting as a function of *R* of (**e**, **f**) Rb_3_Y(PO_4_)_2_:8%Eu and (**g**, **h**) Rb_3_Y(PO_4_)_2_:40%Eu. The gray dashed area represents the data fitting area. **i** Schematic diagram of the luminescence mechanism for Rb_3_Y(PO_4_)_2_:Eu phosphors with different Eu concentrations. The thickness of the straight arrow represents the amount of Eu content entering the cation sites

The photoluminescence (PL) and photoluminescence excitation (PLE) spectra of Rb_3_Y(PO_4_)_2_:*x*Eu (*x* = 0.8%, 70%) phosphors at room temperature (RT) are displayed in Fig. 3a. Under 365 nm excitation, Rb_3_Y(PO_4_)_2_:*x*Eu (*x* = 0.8%) phosphor presents a blue-violet emission at 427 nm accompanied by a sub-shoulder peak, and Rb_3_Y(PO_4_)_2_:*x*Eu (*x* = 70%) phosphor shows a green emission at 514 nm, both of which are ascribed to the 5*d* → 4 *f* transition of Eu^2+^ ions. Their asymmetric peaks imply the presence of multiple luminescent centers in these two phosphors. The PLE spectra of the two samples monitored at their respective optimal wavelengths both exhibit broad bands, ranging from 230–410 nm for *x* = 0.8% phosphor and 230–450 nm for *x* = 70% phosphor, indicating that they can be excited by commercial near-ultraviolet (*n*-UV) chips. The diffuse reflectance spectra of Rb_3_Y(PO_4_)_2_ host and Rb_3_Y(PO_4_)_2_:*x*Eu (*x* = 0.8%, 70%) phosphors are shown in Supplementary Fig. S5, indicating the excitation peak at 270 nm comes from the Rb_3_Y(PO_4_)_2_ host and the other excitation peaks come from Eu^2+^ in Rb_3_Y(PO_4_)_2_. However, because Eu^2+^ occupies multiple cation sites, and the 5*d* energy level of Eu^2+^ is influenced by the host, leading to crystal field splitting, it is difficult to assign excitation peak positions to the crystallographic sites occupied by Eu^2+^. The distinct shapes observed in the PLE spectra of the two samples suggest that the luminescent centers of blue-violet-emitting and green-emitting phosphors are not identical.

To elucidate the relationship between their luminescent centers and crystallographic sites, the emission spectra of Rb_3_Y(PO_4_)_2_:*x*Eu (*x* = 0.8%, 70%) at low-temperature (80 K) were measured, as shown in Fig. 3b, c. For the blue-violet-emitting phosphor (*x* = 0.8%), the PL spectrum at 80 K can be deconvoluted into three Gaussian emission peaks at 23287 cm^−1^ (≈429.4 nm), 21735 cm^−1^ (≈460.1 nm), and 19482 cm^−1^ (≈513.3 nm). Meanwhile, the emission spectrum of the green-emitting phosphor (*x* = 70%) at 80 K is well separated into two Gaussian peaks at 21384 cm^−1^ (≈467.6 nm) and 19745 cm^−1^ (≈506.4 nm). The origin of these emission bands can be analyzed using Van Uitert’s empirical formula^29^, as follows:

$$E=Q\left[1-{\left(\frac{V}{4}\right)}^{\frac{1}{V}}\ast {10}^{-\frac{n^{\ast} EA^{\ast} r}{80}}\right]$$

where *E* (cm^−1^) refers to the emission peak position, *Q* represents the position of the free ions at the lowest energy *d*-band edge (*Q* = 4.2 eV = 34000 cm^−1^ for Eu^2+^), *V* is the valence of the activator (*V* = 2 for Eu^2+^), *n* is the coordination of the activator, *EA* (eV) denotes the electron affinity energy of the anion, and *r* (Å) is the radius of the cation substituted by the activator. Therefore, *E* is positive correlation with *n*, *EA* and *r*. Here, *n*(Rb2O_12_) > *n*(Rb1O_7_) > *n*(YO_6_), *EA* has the same values for Rb1, Rb2 and Y, and *r*(Rb2O_12_) = *r*(Rb1O_7_) > *r*(YO_6_), thus *E*(Rb2O_12_) > *E*(Rb1O_7_) > *E*(YO_6_). As a result, the emission peaks at ≈ 430 nm, ≈ 465 nm, and ≈ 510 nm originate from the Eu^2+^ ions occupying the Rb2O_12_, Rb1O_7_, and YO_6_ sites, respectively. In summary, the blue-violet emission observed in samples with low Eu-doping concentration arises from Eu^2+^ in the Rb1O_7_ and Rb2O_12_ sites, with a minor contribution from the YO_6_ sites that causes a sub-shoulder peak. On the other hand, the green emission of the samples doped with high Eu concentration is caused by Eu^2+^ in the Rb1O_7_ and YO_6_ sites. Furthermore, we measured the radiative decay curves of Rb_3_Y(PO_4_)_2_:*x*Eu (*x* = 0.8%, 70%) at 80 K. As shown in Supplementary Fig. S6, the decay times (τ) monitored at 430 nm, 460 nm, and 513 nm for the *x* = 0.8% sample are determined to be 0.491 μs, 0.549 μs, and 0.677 μs, respectively. For the *x* = 70% sample, the lifetimes monitored at 468 nm, and 506 nm are measured to be 0.550 μs and 0.621 μs. These distinct radiative lifetimes further confirm the existence of multiple luminescent centers in blue-violet-emitting and green-emitting phosphors. The increase in decay time as the monitored emission wavelength rises can be understood by τ ∝ *n* | χ(*n*) |^2^${{\rm{\lambda }}}_{{\rm{em}}}^{3}$, where *χ*(*n*) is a local field correction factor that depends on the refractive index of the host compound at the given wavelength λ_em_^30,31^. In addition, the emission band of the *x* = 70% sample are different at 80 K and RT. Consequently, the temperature-dependent emission spectra of the phosphor in the range of 80 K – 305 K were measured, as depicted in Supplementary Fig. S7a. The emission peak shifts from 470 nm at 80 K to 515 nm at 305 K (Supplementary Fig. S7b), which may be due to the energy transfer of different Eu^2+^ centers at Rb1O_7_ and YO_6_ sites. Subsequently, we performed the time-resolved photoluminescence spectroscopy (Supplementary Fig. S7c), which recorded the short-to-long wavelength emission bands from 100 – 1500 ns. As the recording time increases, the emission peak shift from 470 nm at 100 ns to 515 nm at 1500 ns, confirming the presence of Eu(Rb1)-Eu(Y) energy transfer.

To investigate the intrinsic mechanism for the site occupancy with the increase of Eu concentrations, we performed density functional theory (DFT) calculations to estimate the formation energy of different Eu doping configurations, and the simulation results are shown in Supplementary Fig. S8. The modeling of Eu substitutions was simplified where extra vacancies for charge balance were omitted due to the complex configurations. The formation energies of single occupation were calculated for Rb1-Eu, Rb2-Eu, and Y-Eu at low concentrations. It is found that Y-Eu substitution has the highest formation energy, followed by Rb1-Eu substitution, and Rb2-Eu substitution exhibit the lowest formation energy. Theoretically, a lower formation energy indicates that the corresponding atomic configuration represents a thermodynamically more stable state. Therefore, within a relatively low Eu doping amount, Eu^2+^ ions prefer to occupy Rb1 and Rb2 sites, with a lesser preference for Y sites. Meanwhile, we calculated the formation energies of dual substitutions of Eu ions in the cationic sites, including 2Rb1-2Eu, 2Rb2-2Eu, Rb1-Rb2-2Eu, 2Y-2Eu, Rb1-Y-2Eu and Rb2-Y-2Eu. Upon high Eu doping concentrations, it was observed that the formation energy of Rb1Y-2Eu appears to be the lowest, indicating that Eu^2+^ ions prefer to occupy both the Rb1 and Y sites rather than other configurations. The DFT results evidence that the preferential occupation of Eu^2+^ ions is associated with the doping Eu concentrations in this host.

The site occupation of Eu analyzed by spectroscopy and DFT calculations are consistent with the above refinement results. However, the refinement result of Rb_3_Y(PO_4_)_2_:*x*Eu (*x* = 8%) shows that a significant portion of Eu occupies the YO_6_ sites, while only a small amount of Eu^2+^ is present in the YO_6_ site observed from the PL spectrum (Fig. 3b), suggesting that there may be a large amount of Eu^3+^ in the YO_6_ sites. Therefore, we measured the PL spectra of Rb_3_Y(PO_4_)_2_:*x*Eu (*x* = 0.8%, 8%, 40%, 70%) excited at 254 nm, as shown in Supplementary Fig. S9. All samples exhibit sharp emission lines at 593 nm and 612 nm, except for the Eu^2+^ luminescence, which are attributed to the ^5^D_0_→^7^F_1_ magnetic dipole transition and ^5^D_0_ → ^7^F_2_ electric dipole transition of Eu^3+^^32^. Interestingly, the relative intensity of these two sharp peaks changed with increasing Eu concentration. At low Eu doping concentrations (*x* = 0.8%, 8%), the emission intensity of the ^5^D_0_→^7^F_1_ transition is higher than that of ^5^D_0_→^7^F_2_ transition, indicating that Eu^3+^ primarily occupies an inversion symmetry site, namely YO_6_ site. Due to the trivalent nature of Y^3+^ ions, Eu^3+^ is more stable at Y sites and is less likely to be reduced to Eu^2+^ under the present reducing condition (20%H_2_/80%N_2_). When the location of the Eu^3+^ ion lacks inversion symmetry, the ^5^D_0_→^7^F_2_ electric dipole transition becomes dominant with a notable increase in emission intensity. Thus, the Eu^3+^ ions in the samples doped with high Eu concentration predominantly occupy the low symmetry Rb1 sites. The coexistence of Eu^2+^ and Eu^3+^ in Rb_3_Y(PO_4_)_2_:Eu phosphors is further demonstrated by magnetic susceptibility measurement and Eu *L*_3_-edge X-ray absorption near-edge structure (XANES). The temperature (T) dependence of the molar magnetic susceptibility (χ) and the inverse molar magnetic susceptibility (1/χ) of Rb_3_Y(PO_4_)_2_:*x*Eu (*x* = 8%, 40%) are shown in Supplementary Fig. S10. The valence of Eu ions has a significant impact on the magnetic properties of materials, with Eu^2+^ materials exhibiting notable paramagnetism compared to Eu^3+^ materials^33^. For paramagnetic materials, there is a linear relationship between 1/χ and T, following the Curie-Weiss law. Thus, the linear sections in Supplementary Fig. S10b-c are due to the presence of Eu^2+^, while the curved sections are due to the presence of Eu^3+^^34^. It can be observed that Rb_3_Y(PO_4_)_2_:*x*Eu (*x* = 8%, 40%) samples contain a significant amount of Eu^3+^ and the sample of *x* = 8% exhibits a higher proportion of Eu^3+^ compared to the sample of *x* = 40%. Despite the presence of a large amount of Eu^3+^ in the samples, the Eu^2+^ luminescence can be clearly visualized since 5*d*→4 *f* transition of Eu^2+^ belongs to a parity-allowed electrical dipole transition. On the other hand, the Eu-*L*_3_ near-edge absorption spectra of Rb_3_Y(PO_4_)_2_:*x*Eu (*x* = 0.8%, 8%, 40%, 70%) samples exhibit two absorption peaks at 6974 eV and 6983 eV (Fig. 3d), corresponding to the 2*p*_3/2_→5*d* energy level transitions of Eu^2+^ and Eu^3+^, which further verify that the green-emitting samples (*x* = 40%, 70%) have a higher concentration of Eu^2+^ compared to the blue-violet-emitting samples (*x* = 0.8%, 8%)^12,35^. Therefore, Eu ions are more easily reduced in the green-emitting phosphors thereby leading to higher luminescence intensity. This is understandable because Eu^2+^ occupies both the Rb1 and Y sites in the high Eu doping concentration samples (*x* > 15%), resulting in a balanced charge distribution (Rb^+^ + Y^3+^ → 2Eu^2+^) that enhances the stability of Eu^2+^ ions in the lattice. Subsequently, the local structures of Eu ions in the Rb_3_Y(PO_4_)_2_ lattice are further verified by Eu *L*_3_-edge extended X-ray absorption fine structure (EXAFS) measurement and analysis. To ensure detection sensitivity and minimize the effects of impurities, two typical Eu doping concentrations of *x* = 8% and 40% were chosen for the analysis. The *k*^3^-weighted Eu *L*_3_-edge EXAFS spectra and corresponding Fourier transform fitting as a function of *R* are presented in Fig. 3e-h, and the fitting parameters are shown in Supplementary Table S5. It is worth noting that the fitted results include the Eu^2+^ and Eu^3+^ ions, and *R* represents the distance between atoms, which does not indicate that the atoms are bonded. In the structure of Rb_3_Y(PO_4_)_2_, the coordination numbers of Rb1, Rb2 and Y are 7, 12 and 6, respectively; the average bond lengths of Rb1-O, Rb2-O, and Y-O are 2.9021 Å, 3.3235 Å, and 2.2737 Å, respectively. For the Rb_3_Y(PO_4_)_2_:*x*Eu (*x* = 8%) phosphor, the fitted coordination number (*C*.*N*.) of 7.2±3.2 indicates the presence of Eu ions in the Rb1 sites. Actually, Eu also occupies the Rb2 sites, but the fitted *C*.*N*. is less than the Rb2 coordination number of 12. Because the Y sites are occupied by a certain amount of Eu^3+^ ions, resulting in the small *C*.*N*. and *R*. On the other hand, for the Rb_3_Y(PO_4_)_2_:*x*Eu (*x* = 40%) phosphor, we fitted two Eu-O paths, as shown in Fig. 3h. For the strongest amplitude, the fitted *C*.*N*. (5.7±1.1) is similar to the Rb1 and Y coordination numbers, and the fitted *R* (2.28±0.01 Å) is between the Rb1-O and the Y-O bond length, which confirms that Eu ions occupy the Rb1 and Y sites except for Rb2 site. For the second significant amplitude, the fitted *C*.*N*. (6.8 ± 2.2) is similar to the Rb1 coordination number, but the fitted *R* (3.73 ± 0.02 Å) is larger than the average bond lengths of Rb1-O, Rb2-O and Y-O. Due to the relatively long distance of 3.7 Å between Eu and O, it may be the distance between Eu and O of the second shell layer.

Based on the analysis and demonstration above, Fig. 3i presents the schematic diagram illustrating the luminescence mechanisms of Rb_3_Y(PO_4_)_2_:Eu phosphors. The samples with low Eu-doping concentration (*x* = 0–15%) contain a significant amount of Eu^3+^, which mainly occupies the Y sites, with the remaining small amount of Eu^2+^ occupying the Rb1, Rb2 and Y sites. This overall causes an increase in cell volumes *V* (Part I of Fig. 2e). As the Eu content continues to increase, more Eu^3+^ ions are reduced to Eu^2+^ ions, which occupy the Rb1 and Y sites. In contrast, Eu^3+^ mostly occupies the Rb1 sites. For high Eu-doping concentration (*x* = 20%–100%), the variation of in *V* can be divided into three parts. First, when *x* ranges from 20% to 60%, the increase in *V* due to the substitution of Eu^2+^ for Y^3+^ is largely offset by the decrease in *V* due to the substitution of Eu^2+^ and Eu^3+^ for Rb^+^, leading to a very slow increase in *V* (Part II of Fig. 2e). Then, as *x* increases from 60% to 90%, a large amount of Eu^2+^ occupies the Y site, resulting in a dramatic increase in *V* (Part III of Fig. 2e). Lastly, from 90% to 100% of *x*, very little Eu enter the lattice, so *V* is almost unchanged (Part IV of Fig. 2e). The trend of the variation of the cell volumes *V* in Rb_3_Y(PO_4_)_2_:*x*Eu (*x* = 0-100%) phosphors provides valuable insight into the distribution of Eu ions in the Rb_3_Y(PO_4_)_2_ lattice.

### White LED application

**Fig. 4:**
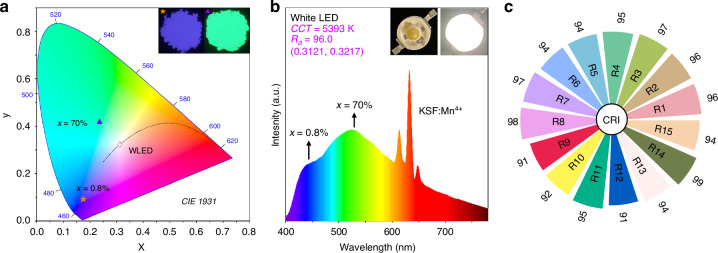
**White LED application of Rb**_**3**_**Y(PO**_**4**_**)**_**2**_**:Eu phosphors. a** CIE color coordinates of Rb_3_Y(PO_4_)_2_:*x*Eu (*x* = 0.8%, 70%) phosphors and the as-fabricated white LED device. The insets are photographs of the luminescence of these phosphors under a 365 nm lamp. **b** The emission spectra of white LED fabricated by the blue-violet-emitting Rb_3_Y(PO_4_)_2_:*x*Eu (*x* = 0.8%) phosphor, the green-emitting Rb_3_Y(PO_4_)_2_:*x*Eu (*x* = 70%) phosphor, commercial red-emitting KSF:Mn^4+^ phosphor and an *n*-UV LED chip (*λ* = 375 nm) with a current of 20 mA. The insets show the photographs of the white LED. **c** The color render index (CRI) of the white LED device

To evaluate the practical performance of the obtained phosphors for LED applications, Rb_3_Y(PO_4_)_2_:*x*Eu (*x* = 0.8%, 70%) were utilized to construct a white LED device with the commercial phosphors and *n*-UV LED chips (*λ* = 375 nm). The corresponding CIE chromaticity diagram calculated from the PL spectra of the two phosphors and their luminescence photographs are shown in Fig. 4a. Moreover, Rb_3_Y(PO_4_)_2_:Eu phosphors exhibit good chemical stability, because the emission intensity of the two phosphors is basically unchanged after being exposed to the ambient atmosphere for 7 days (Supplementary Fig. S11). The blue-violet-emitting Rb_3_Y(PO_4_)_2_:0.8%Eu and green-emitting Rb_3_Y(PO_4_)_2_:70%Eu phosphors can be combined with the commercial red phosphor K_2_SiF_6_:Mn^4+^ (KSF:Mn^4+^) and an *n*-UV LED chip (*λ* = 375 nm) to fabricate the LED device, as presented in Fig. 4b. The LED device emits a bright white light with a correlated color temperature (CCT) of 5393 K, and the CIE color coordinate of (0.3121, 0.3217) is located on the Planckian locus (Fig. 4a). Furthermore, the white LED has an excellent color render index with a high *R*1-*R*15 (Fig. 4c), resulting in a high *R*_*a*_ of 96, which can meet the demands of full-spectrum lighting. For comparison, we fabricated a white LED (blue LED chip + Y_3_Al_5_O_12_:Ce^3+^ + KSF:Mn^4+^) with similar CCT (5393 K), and the normalized emission spectra of the two white LEDs are shown in Supplementary Fig. S12. Because of the cyan gap between the blue and yellow peaks in the 470-510 nm region, the *R*_*a*_ is only 80.7, which is lower than the *R*_*a*_ of 96 achieved by the as-fabricated white LED. Furthermore, we compared the performance of various white LED reported in papers with the as-fabricated white LED, as presented in Supplementary Table S6. The *R*_*a*_ of most white LEDs fabricated by blue, green, and red phosphors with *n*-UV chips are lower than *R*_*a*_ of the as-fabricated white LED, and some even require the use of more than three types of phosphors. The above results indicate that Rb_3_Y(PO_4_)_2_:*x*Eu (*x* = 0.8%, 70%) phosphors can be applied to full-spectrum lighting as the blue and green components, respectively.

## Discussion

In summary, we present a design principle for elucidating concentration quenching by investigating the connectivity and distance among activators in Eu^2+^ doped phosphors. The varying concentration quenching rates in Rb_3_Y(PO_4_)_2_:Eu phosphor were compared by manipulating the occupation of Eu at different lattice sites, thereby providing a comprehensive understanding of the mechanism for suppressing concentration quenching to achieve high luminescence efficiency. At low Eu doping concentrations of 0.1%-15%, Eu^2+^ occupy three cationic sites (Rb1O_7_, Rb2O_12_ and YO_6_). The probability of energy migration increases due to the direct or indirect connection of the cations, leading to a blue-violet luminescence quenching at very low Eu doping concentrations (0.8%). At high Eu concentrations of 20%–100%, Rb_3_Y(PO_4_)_2_:Eu phosphors exhibit green emission due to the preferential occupation of Rb1 and Y sites by Eu^2+^. The large interionic distances and the lack of direct connection between Rb1O_7_-Rb1O_7_ and YO_6_-YO_6_ greatly reduce the rate of concentration quenching, which occurs at Eu concentrations as high as 70%. Finally, the white LED device was fabricated to demonstrate the potential application for full-spectrum lighting. These results suggest the beneficial role of the multiple cationic lattice sites and large interionic distances in alleviating concentration quenching, allowing for the achievement of Eu^2+^-activated phosphors with high luminescence efficiency.

## Materials and methods

### Synthesis

Rb_3_Y(PO_4_)_2_ samples doped with different concentrations of Eu (ranging from 0% to 100%) were synthesized using the high-temperature solid-state reaction method. The raw materials of Rb_2_CO_3_ (99.99%, Aladdin), Y_2_O_3_ (99.99%, Aladdin), NH_4_H_2_PO_4_ (99%, Aladdin), and Eu_2_O_3_ (99.99%, Aladdin) were weighed in stoichiometric ratios and homogeneously ground for about 30 min. The mixtures were pre-sintered in a chamber furnace at 700 °C for 2 h and then ground into powders. The as-prepared precursors were transferred into alumina crucibles and sintered at 1300 °C for 4 h under a reducing atmosphere of 80%N_2_/20%H_2_ in a tube furnace. The obtained products were naturally cooled to room temperature (RT) and reground for further characterization.

### Characterization

The powder X-ray diffraction (XRD) data were recorded by PANalytic X-Pert diffractometer, operating at 45 kV and 40 mA with monochromatized Cu Kα radiation. The Rietveld refinement analysis was performed using Fullprof software. High-angle annular dark-field imaging (HAADF)-scanning transmission electron (STEM) and energy-dispersive X-ray spectroscopy (EDS) mapping images were obtained by FEI Titan Themis G3 operated at 300 kV. The photoluminescence (PL) and photoluminescence excitation (PLE) at RT were collected by FLS1000 fluorescence spectrophotometer (Edinburgh Instruments Ltd., U.K.) with a 450 W Xe900 lamp as the excitation source. The low-temperature (80 K, cooled by liquid nitrogen) PL spectra were measured by FLS1000 with an Oxford Instrument. The decay curves and time-resolved emission spectroscopy (TRES) were measured by FLS1000 with a 375 nm pulse laser diode as the excitation source. Magnetization measurements were performed with an MPMS3 (Quantum Design) at applied fields (*H*) up to 1000 Oe. The X-ray absorption fine structure (XAFS), including X-ray absorption near edge structure (XANES) and extended X-ray absorption fine structure (EXAFS) spectra, were obtained at the Beijing Synchrotron Radiation Facility on the 1W1B beamline. The EXAFS data was processed in Athena (version 0.9.26) for background, pre-edge line and post-edge line calibrations. Then Fourier transform (FT) fitting was completed in Artemis (version 0.9.26). The *k*^3^ weighting, *k*-range of 2-11 Å^−1^ and *R* range of 1-3 Å were used for the fitting of Fe foil; *k*-range of 3.5-8.5 Å^−1^ and *R* range of 1-3 Å were used for the fitting of Rb_3_Y(PO_4_)_2_:*x*Eu (*x* = 8% and 40%). Coordination number (*C*.*N*.), bond length (*R*), Debye-Waller factor (*σ*^2^), and *E*_0_ shift (Δ*E*_0_) were fitted without anyone being fixed, constrained, or correlated.

### White LED fabrication

White LED was fabricated with the blue-violet-emitting phosphor Rb_3_Y(PO_4_)_2_:0.8%Eu, the green-emitting phosphor Rb_3_Y(PO_4_)_2_:70%Eu, the commercial red-emitting phosphor KSF:Mn^4+^ and a commercial *n*-UV LED InGaN chip (*λ* = 375 nm). The optical properties of the fabricated white LED, including emission spectra, correlated color temperature (CCT), CIE color coordinate, color rendering index (CRI, *R*_*a*_), and luminous efficiency, were measured using an integrating sphere spectroradiometer system (OHSP-350M, Hopoocolor).

### Computational methodology

The geometry optimizations for determining the defect formation energy (*E*_*f*_) were calculated by Vienna ab initio simulation package (VASP) simulation code with a plane-wave energy cutoff of 400 eV^36,37^. For the exchange-correlation functional, Perdew-Burke-Ernzerhof (PBE) generalized gradient approximation (GGA) was selected and the projector augmented wave (PAW) potentials have been used to determine the wavefunctions. In order to compare the occupation preference among the low doping and high doping concentration schemes that might lead to concentration-induced luminescence tuning, a 2 × 2 × 1 Rb_12_Y_4_P_8_O_32_ supercell was established before introducing 1 or 2 Eu dopants into the desired atomic sites, corresponding to the low Eu concentrations and high Eu concentrations, respectively. For instance, the case where one Rb1 site was replaced by Eu is referred as Rb1-Eu in the low concentration scheme, while two Eu replacing one Rb2 and Y site, respectively, is noted as Rb2-Y-2Eu in the high concentration scheme. All the geometry optimization convergence energy tolerance was set to 0.05 eV/Å, while the self-consistent field (SCF) for regulating the electronic minimization was set to 5 × 10^−4 ^eV. A 3 × 3 × 3 Monkhorst pack *k*-mesh was employed to sample the Brillouin zone. Based on the optimization results, *E*_*f*_ can be then calculated by the following equation:

$${E}_{f}={{E}_{{tot}}\left({defect}\right)-E}_{{tot}}\left({pristine}\right)-\sum _{i}{n}_{i}{\mu }_{i}$$

where ${E}_{{tot}}\left({defect}\right)$ and ${E}_{{tot}}\left({pristine}\right)$ are the total energy of the corresponding defected model and the perfect cell, respectively. $\sum _{i}{n}_{i}{\mu }_{i}$ represents the total energy variations in the chemical potentials induced by the formation of defects, where ${\mu }_{i}$ is the referencing chemical potential of the corresponding element in its conventional phase (${\mu }_{{Eu}}$ is -4.27 eV from *bcc* Eu, ${\mu }_{{Rb}}$ is -2.31 eV for *bcc* Rb, and ${\mu }_{Y}$ is -9.12 eV from *hcp* Y), and ${n}_{i}$ is the number of different atoms between the defected and perfect structures.

## Supplementary information

LSA20240799-supplementary information for the publication
