# Supplementary material for: Suppressed concentration quenching and tunable photoluminescence in Eu2+-activated Rb3Y(PO4)2 phosphors for full-spectrum lighting
Source: Light Sci Appl. 2024 Sep 20;13:266. doi: 10.1038/s41377-024-01607-x (PMC11415514; doi:10.1038/s41377-024-01607-x)
Supplement: Supplementary file 1 — LSA20240799-supplementary information for the publication [file 41377_2024_1607_MOESM1_ESM.docx]

**Supplementary Information**

**Suppressed Concentration Quenching and Tunable Photoluminescence in Eu^2+^-Activated Rb_3_Y(PO_4_)_2_ Phosphors for Full-Spectrum Lighting**

Ming Zhao^1^, Yeping Ge^1^, Yurong Li^2^, Xiaoyan Song^2^, Zhiguo Xia^3*^ and Xinping Zhang^1*^

^1^Institute of Information Photonics Technology, School of Physics and Optoelectronic Engineering, Beijing University of Technology, Beijing 10083, China. ^2^College of Materials Science and Engineering, Key Laboratory of Advanced Functional Materials, Education Ministry of China, Beijing University of Technology, Beijing 10083, China. ^3^State Key Laboratory of Luminescent Materials and Devices, Guangdong Provincial Key Laboratory of Fiber Laser Materials and Applied Techniques, Guangdong Engineering Technology Research and Development Center of Special Optical Fiber Materials and Devices, School of Physics and Optoelectronics, South China University of Technology, Guangzhou 510641, China. ^*^E-mail: [xiazg@scut.edu.cn](mailto:xiazg@scut.edu.cn) (Z.G.X.); [zhangxinping@bjut.edu.cn](mailto:zhangxinping@bjut.edu.cn) (X.P.Z.)


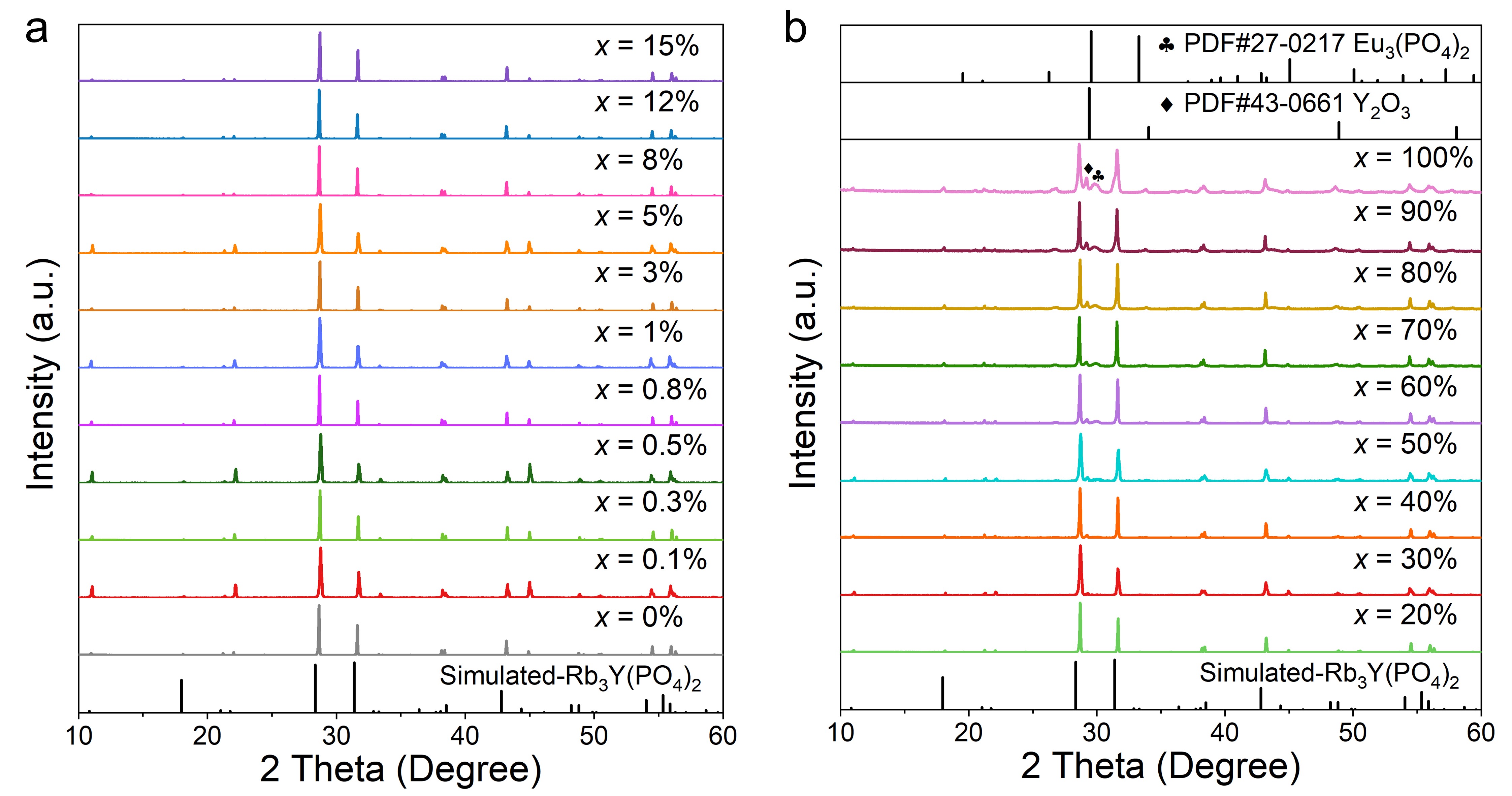


**Figure S1** XRD patterns of Rb_3_Y(PO_4_)_2_:*x*Eu (*x* = 0%-100%) and the simulated pattern of Rb_3_Y(PO_4_)_2_ as a reference.





**Figure S2** Rietveld refinement patterns of Rb_3_Y(PO_4_)_2_:*x*Eu (0% ≤ *x* ≤ 100%).

Note: Because the CIF file of Eu_3_(PO_4_)_2_ is not available, we performed Le Bail fitting for Eu_3_(PO_4_)_2_. Therefore, we cannot refine the exact content of the Eu_3_(PO_4_)_2_ impurity. But this does not affect the analysis of Rb_3_Y(PO_4_)_2_:Eu.


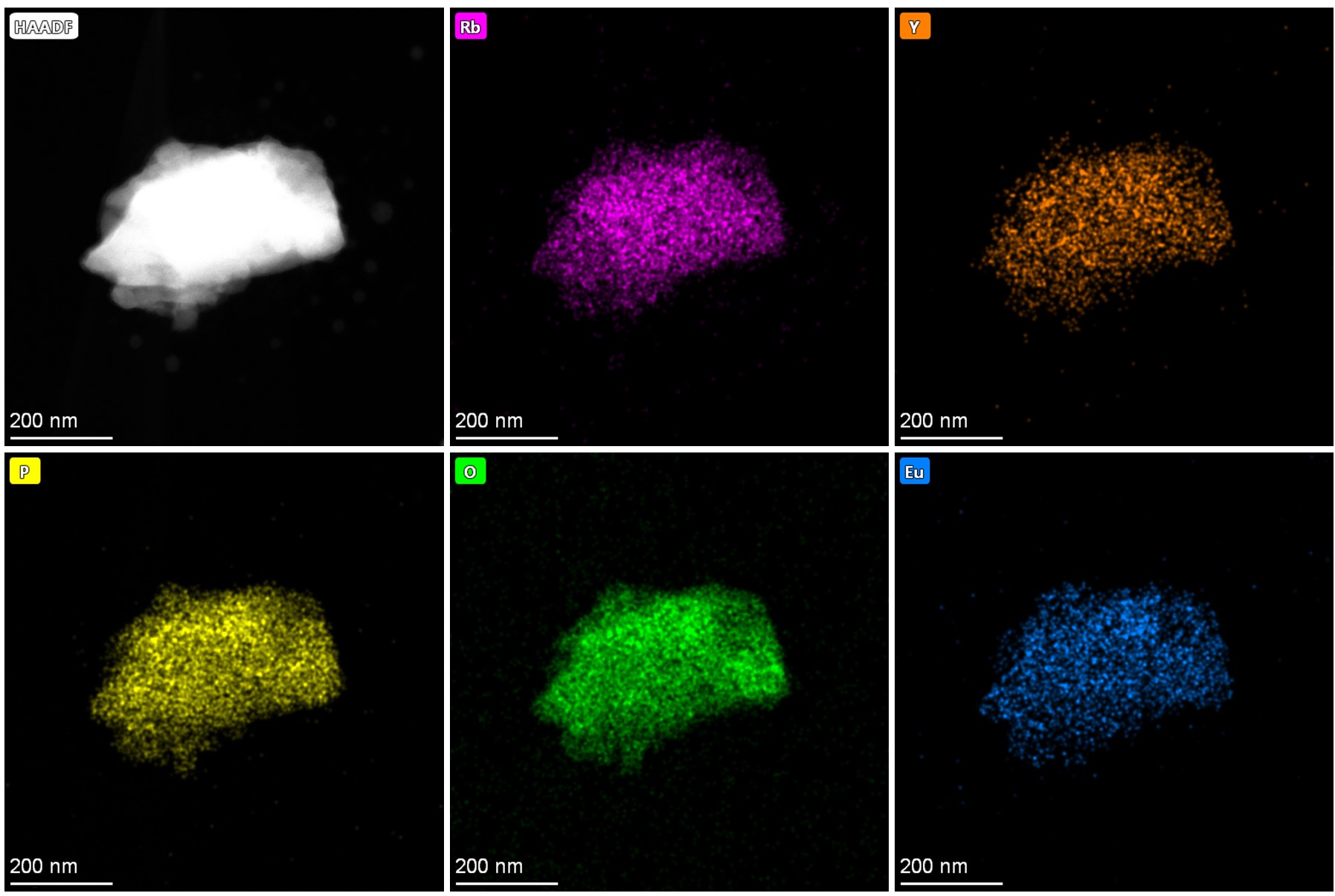


**Figure S3.** HAADF-STEM and EDS-mapping images of Rb_3_Y(PO_4_)_2_:70%Eu.





**Figure S4** The emission spectra of Rb_3_Y(PO_4_)_2_:*x*Eu (8% ≤ *x* ≤ 20%).


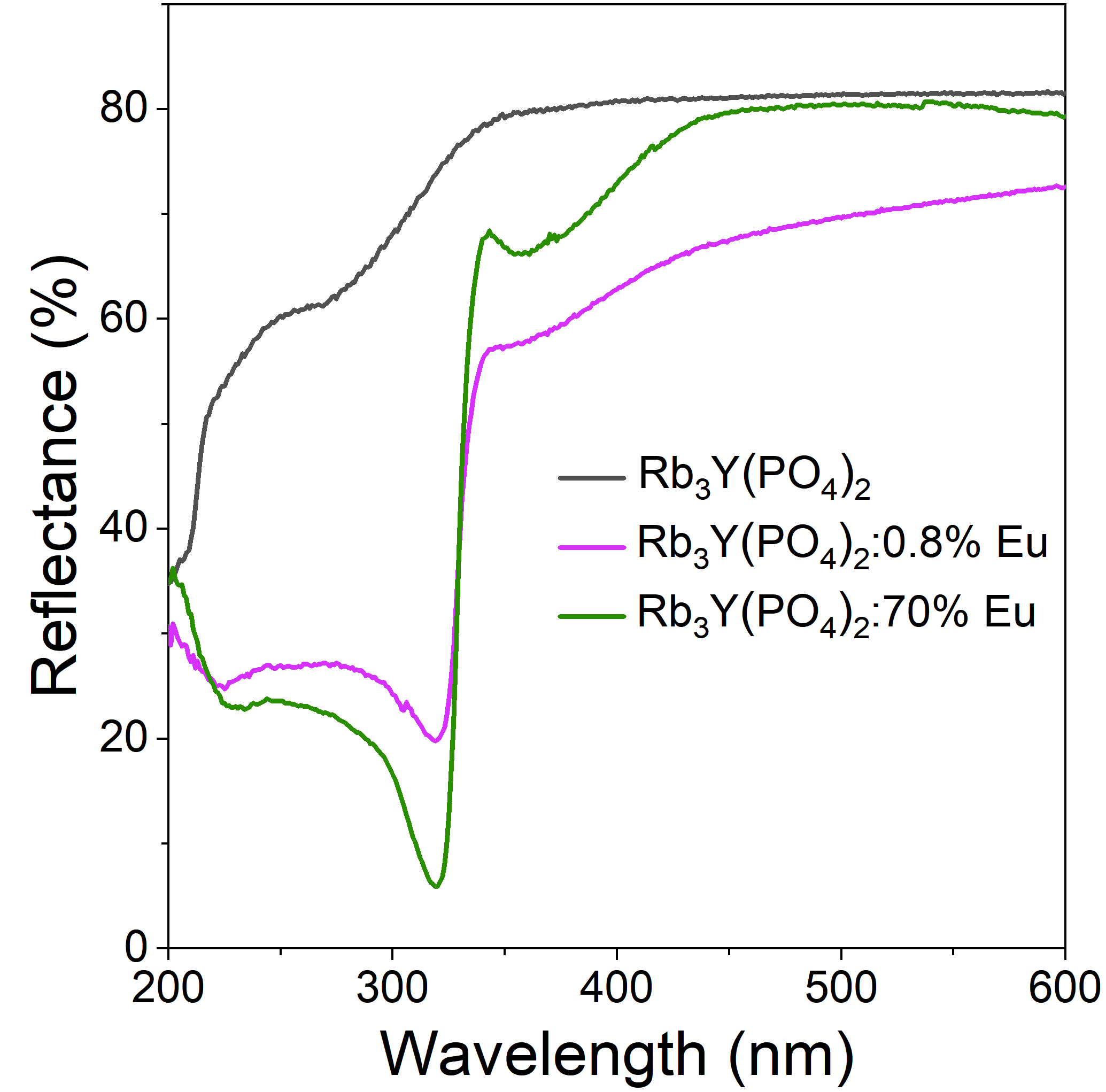


**Figure S5** Diffuse reflectance spectra of Rb_3_Y(PO_4_)_2_ host and Rb_3_Y(PO_4_)_2_:*x*Eu (*x* = 0.8%, 70%) phosphors.


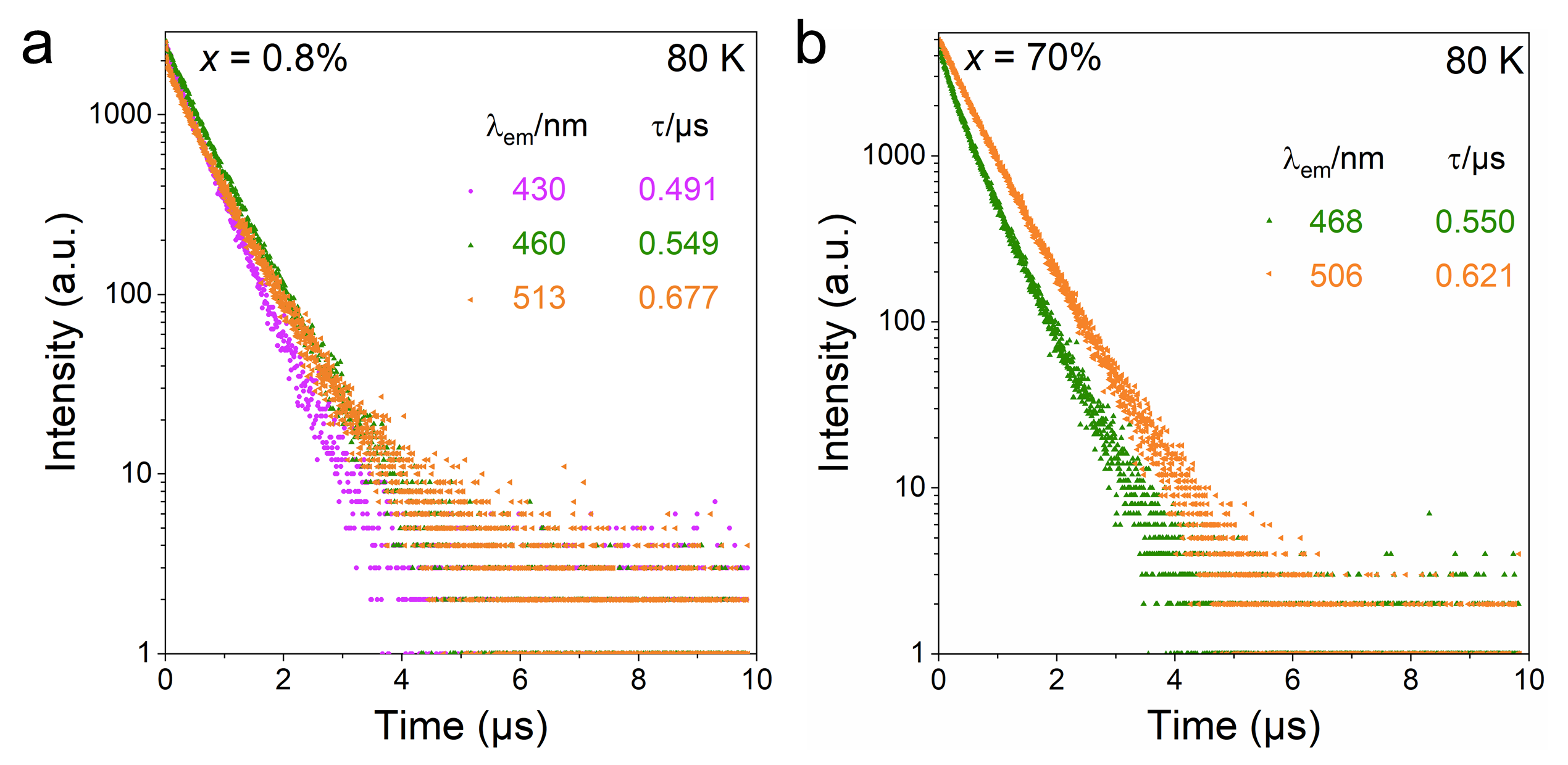


**Figure S6** Decay curves at 80 K of **a** Rb_3_Y(PO_4_)_2_:0.8%Eu and **b** Rb_3_Y(PO_4_)_2_:70%Eu excited at 375 nm pulse laser and monitored at different wavelengths.


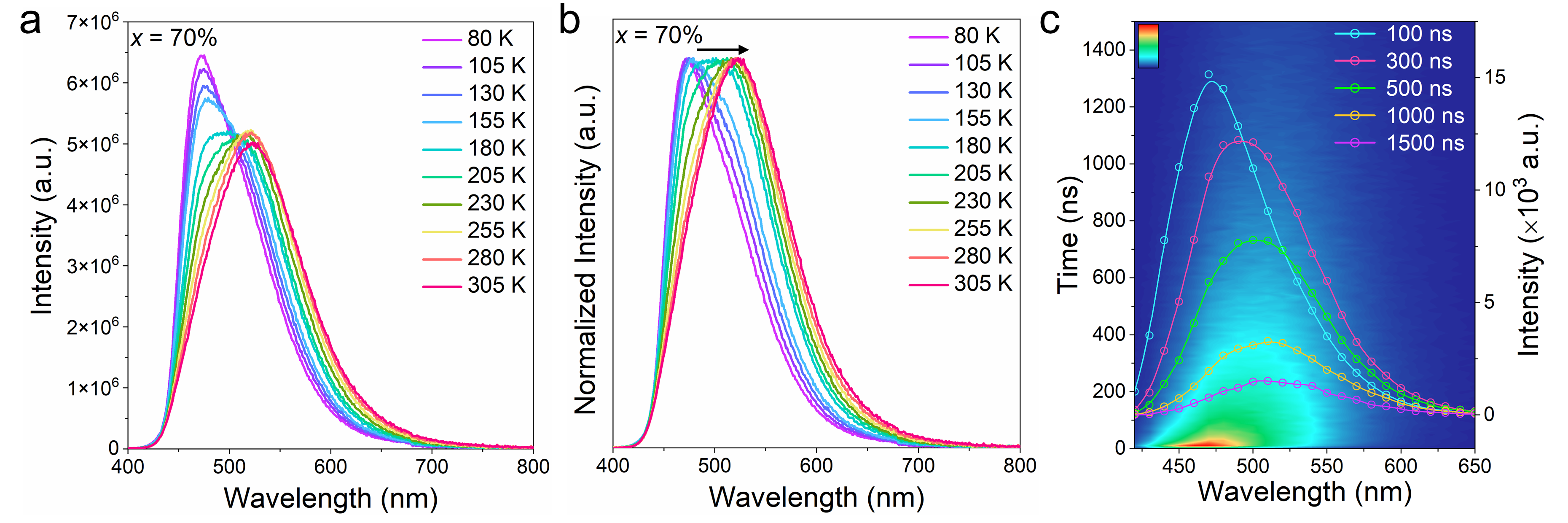


**Figure S7** Temperature-dependent **a** emission spectra and **b** normalized emission spectra of Rb_3_Y(PO_4_)_2_:70%Eu phosphor in the temperature range 80 K-305 K with a temperature interval of 25 K under 365 nm excitation. **c** Time-resolved photoluminescence spectroscopy of Rb_3_Y(PO_4_)_2_:70%Eu under 375 nm pulse laser excitation. The inset spectra are obtained at different delays time.

**
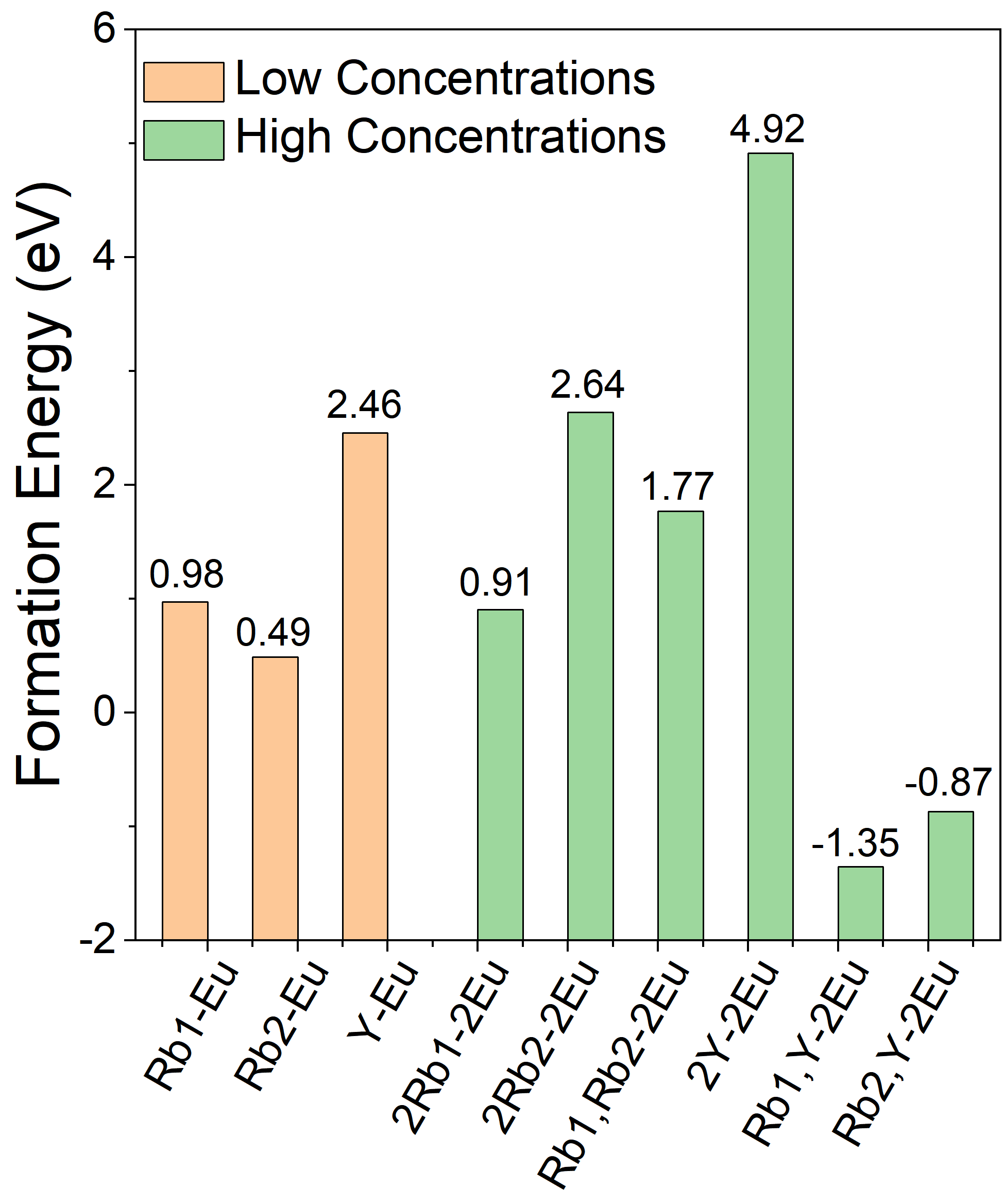
**

**Figure S8** Calculated formation energies for different substitutions of Eu^2+^ in Rb_3_Y(PO_4_)_2_.


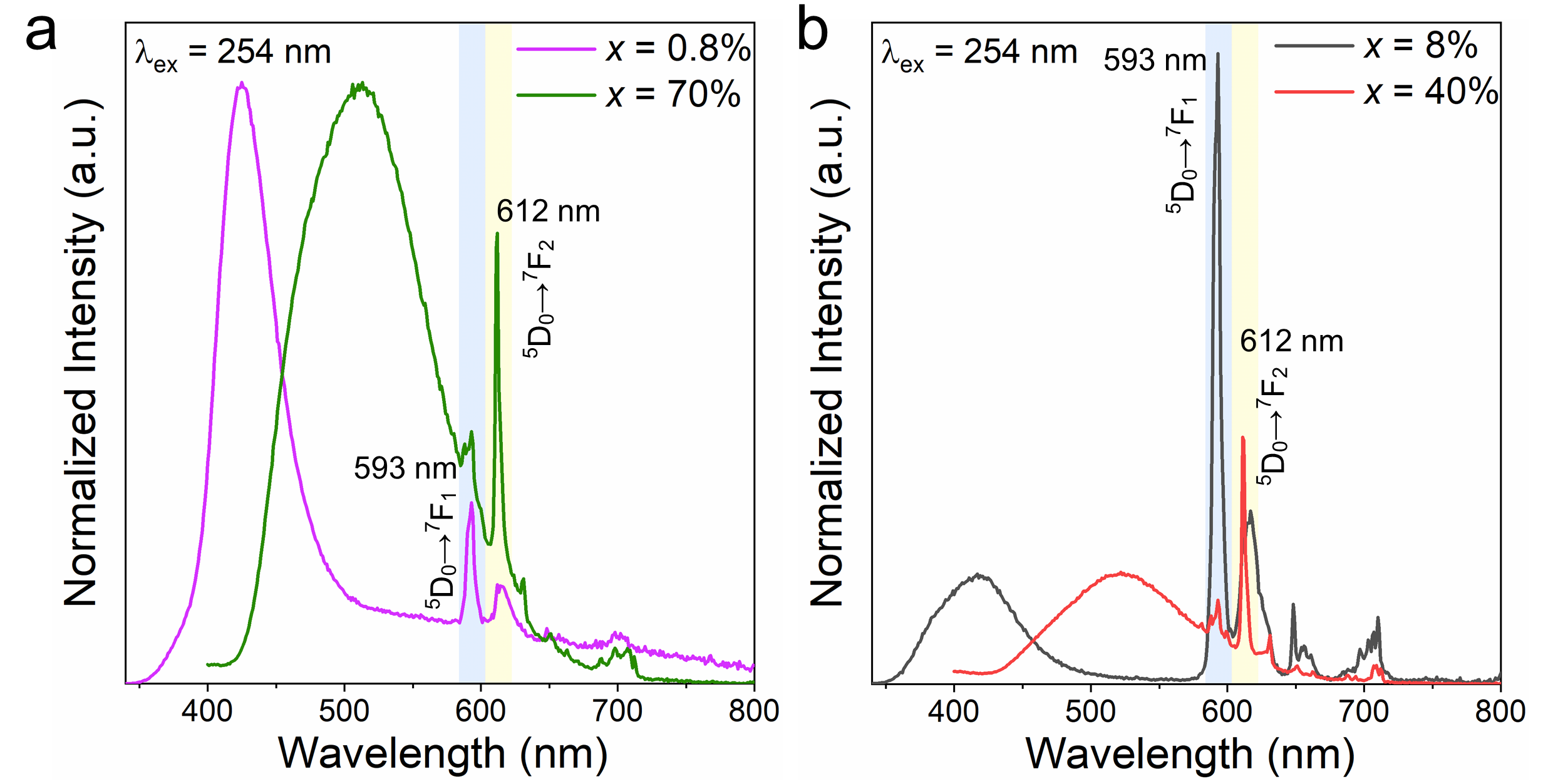


**Figure S9** The emission spectra of **a** Rb_3_Y(PO_4_)_2_:*x*%Eu (*x* = 0.8%, 70%) and **b** Rb_3_Y(PO_4_)_2_:*x*%Eu (*x* = 8%, 40%) under 254 nm excitation.


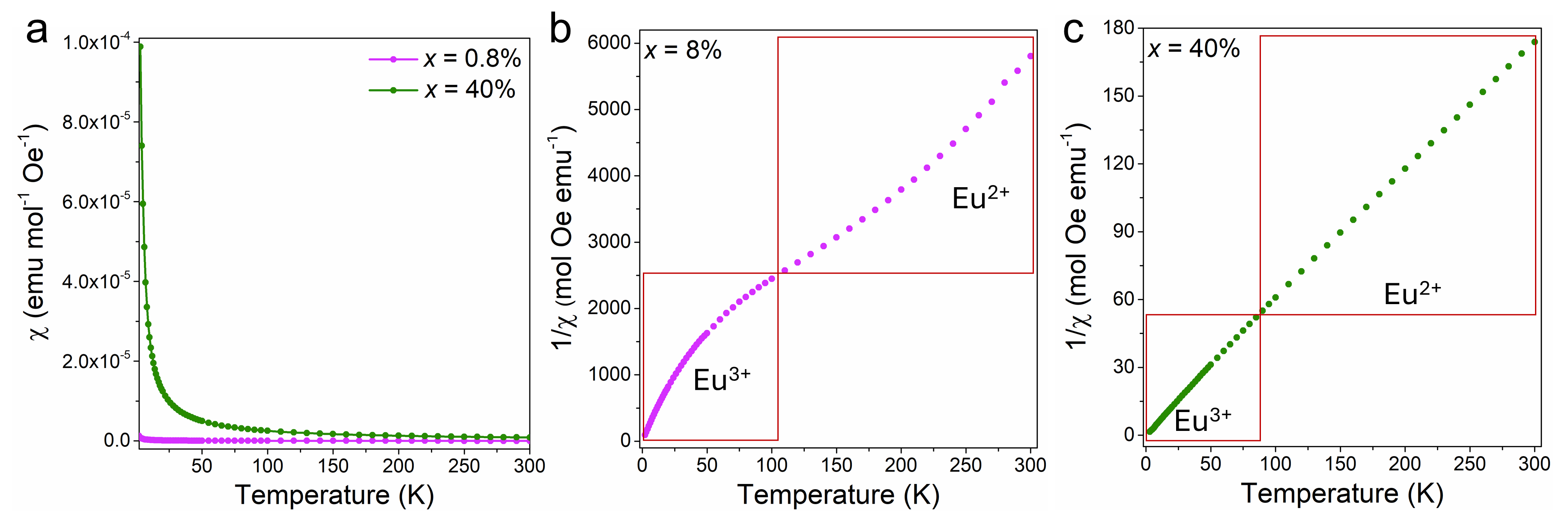


**Figure S10 a**The temperature (T) dependence of the molar magnetic susceptibility (χ) and **b,c** the inverse molar magnetic susceptibility (1/χ) of Rb_3_Y(PO_4_)_2_:*x*Eu (*x* = 8%, 40%).

**
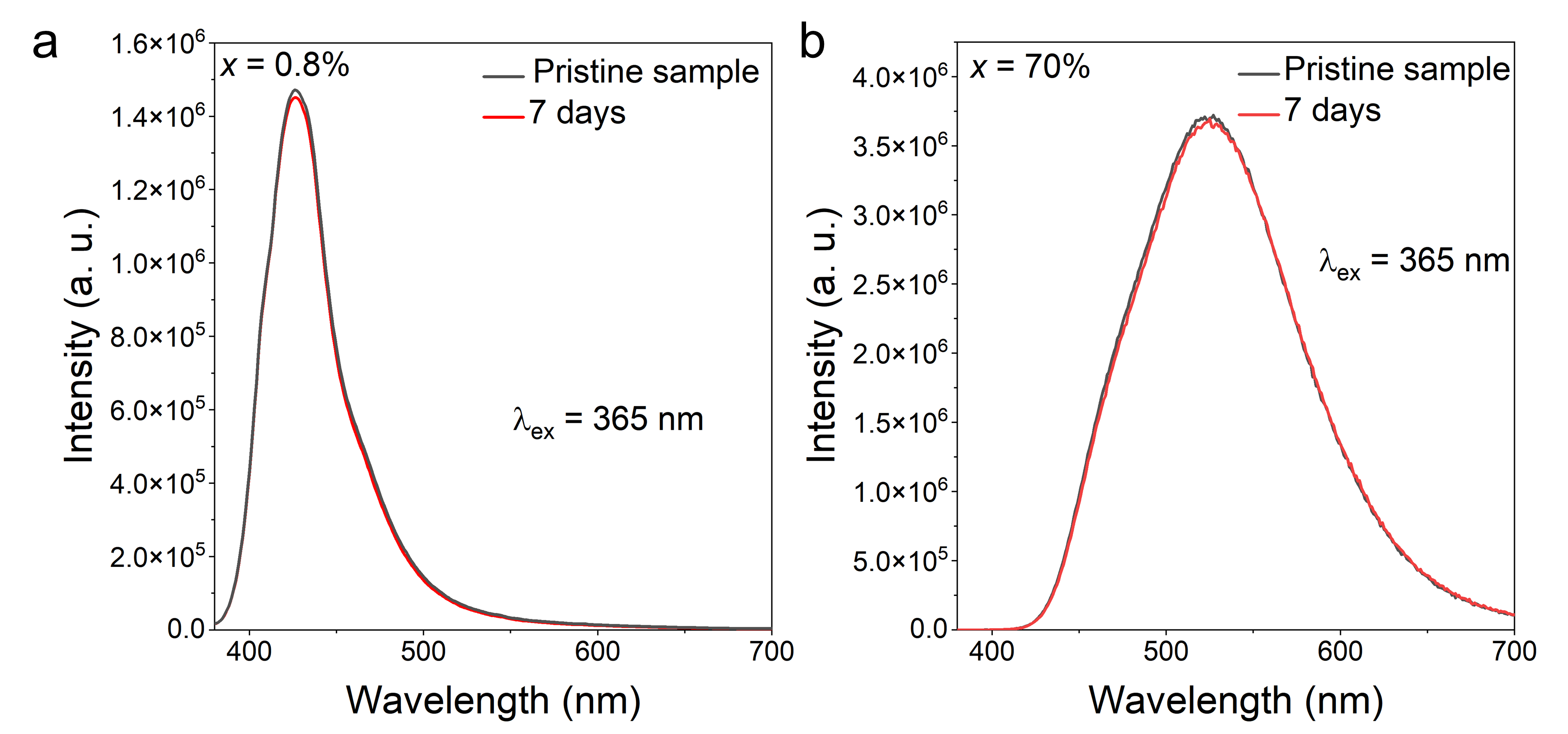
**

**Figure S11** The emission spectra of the pristine Rb_3_Y(PO_4_)_2_:*x*Eu (*x* = 0.8%, 70%) and the samples exposed to ambient atmosphere for 7 days.


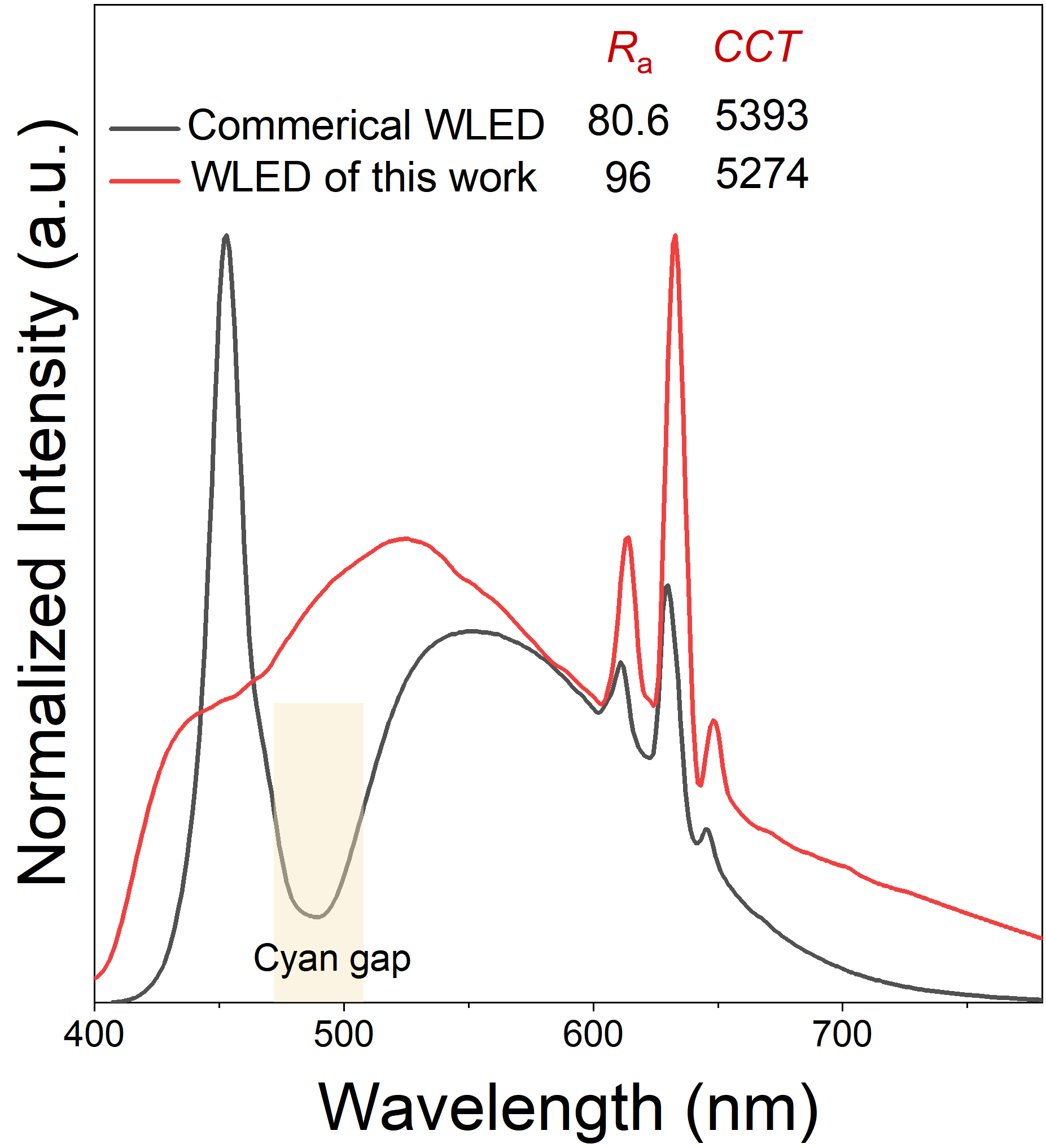


**Figure S12** The normalized emission spectra of the commercial white LED (blue LED chip + Y_3_Al_5_O_12_:Ce^3+^ + K_2_SiF_6_:Mn^4+^) and the as-fabricated white LED of this work.

**Table S1** Radius of Rb, Y and Eu with different coordination numbers.

| Cation | Coordination number | Radius [Å] |
| --- | --- | --- |
| Rb^+^ | 7 | 1.56 |
| Rb^+^ | 12 | 1.72 |
| Y^3+^ | 6 | 0.9 |
| Eu^2+^ | 6 | 1.17 |
| Eu^2+^ | 7 | 1.2 |
| Eu^2+^ | 10 | 1.35 |
| Eu^3+^ | 6 | 0.947 |
| Eu^3+^ | 7 | 1.01 |
| Eu^3+^ | 9 | 1.12 |

**Table S2** Main parameters of processing and refinement of the Rb_3_Y(PO_4_)_2_:*x*Eu (0% ≤ *x* ≤ 100%) samples.

| *x* (%) | Space group | Cell parameters (Å, º),  Cell volume (Å^3^) | *R_p_* (%), *R_wp_* (%)*, R_exp_* (%), *χ*^2^ |
| --- | --- | --- | --- |
| 0 | *Pm*1 | *a* = *b*= 5.65379 (2)  *c* = 8.06739 (2)  *V* = 223.328 (2)  *α*= *β*= 90  *γ* = 120 | 7.80, 11.60, 4.75, 5.95 |
| 0.3 | *Pm*1 | *a* = *b*= 5.65423(2)  *c* = 8.06670 (3)  *V* = 223.344 (2)  *α*= *β* = 90  *γ* = 120 | 11.40, 15.80 8.96, 3.11 |
| 0.8 | *Pm*1 | *a* = *b*= 5.65452 (2)  *c* = 8.06611 (2)  *V* = 223.350 (1)  *α*= *β* = 90  *γ* = 120 | 10.10, 14.60, 8.88, 2.71 |
| 3 | *Pm*1 | *a* = *b*= 5.65500 (2)  *c* = 8.06753 (4)  *V* = 223.507 (2)  *α*= *β* = 90  *γ* = 120 | 9.60, 13.60, 8.49, 2.58 |
| 8 | *Pm*1 | *a* = *b*= 5.65817 (2)  *c* = 8.06818 (3)  *V* = 223.697 (1)  *α*= *β* = 90  *γ* = 120 | 8.97, 12.70, 8.41, 2.29 |
| 12 | *Pm*1 | *a* = *b*= 5.65900 (2)  *c* = 8.06802 (3)  *V* = 223.757 (2)  *α*= *β* = 90  *γ* = 120 | 8.83, 12.40, 8.24, 2.26 |
| 15 | *Pm*1 | *a* = *b*= 5.65965 (1)  *c* = 8.06811 (2)  *V* = 223.811 (1)  *α*= *β* = 90  *γ* = 120 | 9.28, 12.70, 8.22, 2.40 |
| 20 | *Pm*1 | *a* = *b*= 5.66029(1)  *c* = 8.06820(2)  *V* = 223.864(1)  *α*= *β* = 90  *γ* = 120 | 8.99, 12.40, 8.10, 2.34 |
| 40 | *Pm*1 | *a* = *b*= 5.66101 (15)  *c* = 8.06832 (4)  *V* = 223.925 (2)  *α*= *β* = 90  *γ* = 120 | 6.91, 9.52, 5.37, 3.14 |
| 60 | *Pm*1 | *a* = *b*= 5.66146(3)  *c* = 8.06740(7)  *V* = 223.935(2)  *α*= *β* = 90  *γ* = 120 | 6.14, 8.09, 5.20, 2.42 |
| 70 | *Pm*1 | *a* = *b*= 5.66456 (3)  *c* = 8.06978(7)  *V* = 224.246 (3)  *α*= *β* = 90  *γ* = 120 | 4.66, 6.44, 3.65, 3.11 |
| 80 | *Pm*1 | *a* = *b*= 5.66804 (3)  *c* = 8.06903 (10)  *V* = 224.501 (3)  *α*= *β* = 90  *γ* = 120 | 5.16, 7.17, 4.06, 3.12 |
| 90 | *Pm*1 | *a* = *b*= 5.67027 (15)  *c* = 8.0863 (3)  *V* = 225.159 (12)  *α*= *β* = 90  *γ* = 120 | 8.12, 11.7, 3.70, 9.96 |
| 100 | *Pm*1 | *a* = *b*= 5.67029(9)  *c* = 8.0864(2)  *V* = 225.163(9)  *α*= *β* = 90  *γ* = 120 | 7.67, 11.3, 3.63, 9.65 |

**Table S3** Main bond lengths [Å] of Rb_3_Y(PO_4_)_2_:*x*Eu (*x* = 0%, 8%, 40%, 70%).

| *x* = 0% | | | |
| --- | --- | --- | --- |
| Rb1-O1 | 2.946 (6) | Rb2-O2 | 3.339 (17) |
| Rb1-O2 | 2.639 (9) | Y-O1 | 2.274 (4) |
| Rb2-O1 | 3.308 (4) |  |  |
| *x* = 8% | | | |
| Rb1-O1 | 2.949 (7) | Rb2-O2 | 3.338 (2) |
| Rb1-O2 | 2.623 (10) | Y-O1 | 2.288 (5) |
| Rb2-O1 | 3.315 (5) |  |  |
| *x* = 40% | | | |
| Rb1-O1 | 2.950 (10) | Rb2-O2 | 3.367 (4) |
| Rb1-O2 | 2.732 (15) | Y-O1 | 2.286 (8) |
| Rb2-O1 | 3.301 (7) |  |  |
| *x* = 70% | | | |
| Rb1-O1 | 2.933 (8) | Rb2-O2 | 3.398 (4) |
| Rb1-O2 | 3.428 (4) | Y-O1 | 2.257 (7) |
| Rb2-O1 | 3.247 (7) |  |  |

**Table S4** Fractional atomic coordinates of Rb_3_Y(PO_4_)_2_:*x*Eu (*x* = 0%, 8%, 40%, 70%).

| atoms | *x* | *y* | *z* | Occ. | Site |
| --- | --- | --- | --- | --- | --- |
| *x* = 0% | | | | | |
| Y1 | 0 | 0 | 0 | 1 | 1*a* |
| Rb1 | 2/3 | 1/3 | 0.25993(15) | 1 | 2*d* |
| Rb2 | 0 | 0 | 0.5 | 1 | 1*b* |
| P1 | 1/3 | 2/3 | 0.2243(3) | 1 | 2*d* |
| O1 | 0.1904(8) | 0.3808(8) | 0.1613(4) | 1 | 6*i* |
| O2 | 1/3 | 2/3 | 0.4129(10) | 1 | 2*d* |
| *x* = 8% | | | | | |
| Y1 | 0 | 0 | 0 | 0.874 | 1*a* |
| Eu1 | 0 | 0 | 0 | 0.126 | 1*a* |
| Rb1 | 2/3 | 1/3 | 0.2601(2) | 0.97 | 2*d* |
| Eu2 | 2/3 | 1/3 | 0.2601(2) | 0.03 | 2*d* |
| Rb2 | 0 | 0 | 0.5 | 0.97 | 1*b* |
| Eu3 | 0 | 0 | 0.5 | 0.03 | 1*b* |
| P1 | 1/3 | 2/3 | 0.2249(5) | 1 | 2*d* |
| O1 | 0.1918(11) | 0.3836(11) | 0.1616(5) | 1 | 6*i* |
| O2 | 1/3 | 2/3 | 0.4148(12) | 1 | 2*d* |
| *x* = 40% | | | | | |
| Y1 | 0 | 0 | 0 | 0.794 | 1*a* |
| Eu1 | 0 | 0 | 0 | 0.206 | 1*a* |
| Rb1 | 2/3 | 1/3 | 0.2615(3) | 0.834 | 2*d* |
| Eu2 | 2/3 | 1/3 | 0.2615(3) | 0.166 | 2*d* |
| Rb2 | 0 | 0 | 0.5 | 1 | 1*b* |
| P1 | 1/3 | 2/3 | 0.2208(8) | 1 | 2*d* |
| O1 | 0.1908(15) | 0.3816(15) | 0.1629(8) | 1 | 6*i* |
| O2 | 1/3 | 2/3 | 0.3999(18) | 1 | 2*d* |
| *x* = 70% | | | | | |
| Y1 | 0 | 0 | 0 | 0.495 | 1*a* |
| Eu1 | 0 | 0 | 0 | 0.505 | 1*a* |
| Rb1 | 2/3 | 1/3 | 0.2580(4) | 0.704 | 2*d* |
| Eu2 | 2/3 | 1/3 | 0.2580(4) | 0.296 | 2*d* |
| Rb2 | 0 | 0 | 0.5 | 1 | 1*b* |
| P1 | 1/3 | 2/3 | 0.2198(10) | 1 | 2*d* |
| O1 | 0.1850(12) | 0.3700(12) | 0.1663(9) | 1 | 6*i* |
| O2 | 1/3 | 2/3 | 0.3855(16) | 1 | 2*d* |

**Table S5** EXAFS fitting parameters at the Fe *K*-edge and Eu *L*_3_-edge (*Ѕ*_0_^2^ = 0.82) of Rb_3_Y(PO_4_)_2_:*x*Eu (*x* = 8%, 40%).

| Sample | Path | *C.N.* | *R* (Å) | *σ*^2^×10^3^ (Å^2^) | Δ*E* (eV) | *R* factor |
| --- | --- | --- | --- | --- | --- | --- |
| Fefoil | Fe-Fe | 8* | 2.47±0.01 | 4.3±1.9 | 6.5±3.0 | 0.002 |
|  | Fe-Fe | 6* | 2.84±0.02 | 5.3±3.8 | 5.2±4.6 |  |
| *x* = 8% | Eu-O | 7.2±3.2 | 2.31±0.02 | 11.1±4.5 | 3.2±3.0 | 0.016 |
| *x* = 40% | Eu-O | 5.7±1.1 | 2.28±0.01 | 5.1±2.0 | 0.6±2.2 | 0.014 |
|  | Eu-O | 6.8±2.2 | 3.73±0.02 | 1.2±3.1 | 8.2±2.0 |  |

*C.N.*: coordination numbers; *R*: bond distance; *σ*^2^: Debye-Waller factors; Δ*E*: the inner potential correction. *R* factor: goodness of fit. * fitting with fixed parameter.

Note: Due to the low Eu content of the *x* = 8% sample, the data quality is relatively poor, leading to slightly higher errors in the fitting results of *C*.*N*.

**Table S6** Comparison of the performance between various white LEDs reported in papers and the as-fabricated white LED of this work.

| Device Structure | *R_a_* | CCT (K) | CIE coordinates | Refs |
| --- | --- | --- | --- | --- |
| Blue chip/ Y_3_Al_5_O_12_:Ce^3+^ (yellow)/K_2_SiF_4_:Mn^4+^ (red) | 80.6 | 5393 | (0.338, 0.3479) | Commercial white LED |
| *n*-UV chip (365 nm)/BaMgAl_10_O_17_:Eu^2+^ (blue)/Ba_3_La_6_(SiO_4_)_6_:0.05Eu^2+^ (503 nm, green)/CaAlSiN_3_:Eu^2+^ (red) | 82.9 | 4818 | (0.3320, 0.3418) | 1 |
| *n*-UV chip (365 nm)/BaMgAl_10_O_17_:Eu^2+^ (blue) /Sr_2_MgB_2_O_6_:0.05Ce^3+^,0.05Tb^3+^ (544 nm, green)/ K_2_SiF_4_:Mn^4+^ (red) | 85.7 | 5893 | (0.3242, 0.3334) | 2 |
| *n*-UV chip (395 nm)/Na_3_CsMg_7_(PO_4_)_6_:0.03Eu (463 nm, blue)  /(Ba, Sr)SiO_4_:Eu^2+^ (green)/CaAlSiN_3_:Eu^2+^ (red) | 89.7 | 3543 | (0.406, 0.398) | 3 |
| *n*-UV chip (370 nm)/BaMgAl_10_O_17_:Eu^2+^ (blue)/Ba_2_Lu_5_B_5_O_17_:0.01Ce^3+^, 0.15Tb^3+^ (543 nm, green)/CaAlSiN_3_:Eu^2+^ (red) | 91.4 | 3809 | - | 4 |
| *n*-UV chip (400 nm)/BaMgAl_10_O_17_:Eu^2+^ (blue)/Ca_2_LaHf_2_Al_3_O_12_:0.05Ce^3+^ (515 nm, green)/CaAlSiN_3_:Eu^2+^ (red) | 92.0 | 3623 | (0.405, 0.404) | 5 |
| *n*-UV chip (370 nm)/SrZnSO:0.03Bi^3+^ (460 nm, blue)/(Ba, Sr)SiO_4_:Eu^2+^ (green)/(Ca, Sr)AlSiN_3_:Eu^2+^ (red) | 93.4 | 4051 | (0.3817, 0.3885) | 6 |
| *n*-UV chip (400 nm)/Na_3_KMg_7_(PO_4_)_6_:0.04Eu^2+^ (446 nm, blue)  /β-SiAlON:Eu^2+^ (green)/Sr_2_Si_5_N_8_:Eu^2+^ (red) | 94.3 | 3700 | - | 7 |
| *n*-UV chip (400 nm)/BaMgAl_10_O_17_:Eu^2+^ (blue) /Ca_2_GdHf_2_Al_3_O_12_:0.04Ce^3+^, 0.4Tb^3+^ (543 nm, green) /CaAlSiN_3_:Eu^2+^ (red) | 94.4 | 3575 | (0.391, 0.360) | 8 |
| *n*-UV chip (365 nm)/BaMgAl_10_O_17_:Eu^2+^ (blue)/Y_3_Al_5_O_12_:Ce^3+^ (yellow)/CaAlSiN_3_:Eu^2+^ (red) | 94.6 | - | (0.3706, 0.3853) | 9 |
| *n*-UV chip (365 nm)/SrSc_4_Si_5_O_17_:0.03Ce^3+^ (425 nm, blue-violet) /(Ba,Ca,Mg)_2_SiO_4_:Eu^2+^ (475 nm, blue-cyan)  /(Ba,Sr)_2_SiO_4_:Eu^2+^ (green)/Sr_3_SiO_5_:Eu^2+^ (orange)/CaAlSiN_3_:Eu^2+^ (red) | 95.9 | 4661 | (0.1586, 0.0263) | 10 |
| *n*-UV chip (365 nm)/Ba_1.31_Sr_3.69_(BO_3_)_3_Cl:0.03Ce^3+^ (400 nm, blue-violet)  /BaMgAl_10_O_17_:Eu^2+^ (blue)/Y_3_Al_5_O_12_:Ce^3+^ (yellow)/CaAlSiN_3_:Eu^2+^ (red) | 97.2 | - | (0.3436, 0.3539) | 9 |
| *n*-UV chip (375 nm)/Rb_3_Y(PO_4_)_2_:0.8%Eu (427 nm, blue-violet) /Rb_3_Y(PO_4_)_2_:70%Eu (514 nm, green)/K_2_SiF_4_:Mn^4+^ (red) | 96.0 | 5393 | (0.3121, 0.3217) | **this work** |

**References**

1. Zhong, J. et al. Synthesis and spectroscopic investigation of Ba_3_La_6_(SiO_4_)_6_:Eu^2+^ green phosphors for white light-emitting diodes. *Chem. Eng. J* **309**, 795-801 (2017).
2. Dong, Q. et al. A narrow-band ultra-bright green phosphor for LED-based applications. *Dalton T.* **49**, 1935-1946 (2020).
3. Chen, H. et al. An efficient blue phosphor with high thermal stability for lighting and optical pressure sensor applications. *Inorg. Chem. Front.* **9**, 1644-1654 (2022).
4. Xiao, Y. et al. An efficient green phosphor of Ce^3+^ and Tb^3+^-codoped Ba_2_Lu_5_B_5_O_17_ and a model for elucidating the high thermal stability of the green emission. *J. Mater. Chem. C* **6**, 5984-5991 (2018).
5. Liang, J. et al. Filling the cyan gap toward full-visible-spectrum LED lighting with Ca_2_LaHf_2_Al_3_O_12_:Ce^3+^ broadband green phosphor. *J. Alloys Compd.* **836**, 155469 (2020).
6. Dong, Q. et al. Bismuth activated blue phosphor with high absorption efficiency for white LEDs. *J. Alloys Compd.* **885**, 160960 (2021).
7. Leng, Z. et al. A Zero-Thermal-Quenching Blue Phosphor for Sustainable and Human-Centric WLED Lighting. *ACS Sustainable Chem. Eng.* **10**, 10966-10977 (2022).
8. Huang, X. et al. Ultra-high color rendering warm-white light-emitting diodes based on an efficient green-emitting garnet phosphor for solid-state lighting. *Chem. Eng. J* **405**, 126950 (2021).
9. Wu, X. et al. An efficient blue–violet phosphor: an advanced material designed for high-quality full-spectrum lighting. *Dalton T.* **53**, 4564-4573 (2024).
10. Shao, Y. et al. Efficient Blue–Violet Phosphor with Small Stokes-Shift for Full-Spectrum Lighting. *Laser Photonics Rev.* **17**, 2300342 (2023).
